# Healthy Singleton Pregnancies From Restorative Reproductive Medicine (RRM) After Failed IVF

**DOI:** 10.3389/fmed.2018.00210

**Published:** 2018-07-31

**Authors:** Phil C. Boyle, Theun de Groot, Karolina M. Andralojc, Tracey A. Parnell

**Affiliations:** ^1^International Institute for Restorative Reproductive Medicine, London, United Kingdom; ^2^NeoFertility Clinic, Dublin, Ireland; ^3^Department of Molecular Biology, Faculty of Science, Radboud Institute for Molecular Life Sciences, Radboud University, Nijmegen, Netherlands; ^4^Department of Family Medicine, University of British Columbia, Vancouver, BC, Canada

**Keywords:** infertility, restorative reproductive medicine, RRM, IVF failure, fertility treatment, prevention premature birth, prevention multiple births, IVF alternative

## Abstract

**Objectives:** To determine the live birth rate for patients who chose to undergo treatment with Restorative Reproductive Medicine (RRM) after previous IVF (includes ICSI). To look at birth outcomes with RRM after IVF, particularly rates of twin and higher order pregnancies, premature birth, low birth weight, and potential cost savings achieved with RRM.

**Setting:** Two outpatient clinics in Ireland providing advanced RRM treatment of infertility.

**Materials and methods:** All patients presenting between January 2004 and January 2010, with a history of infertility and previous IVF treatment were included if they proceeded beyond the initial consultation and began treatment. Main outcome is live birth per couple calculated using life table analysis.

**Results:** 403 patients met the study criteria, among which 74 had a subsequent live birth. These women had significant negative predictive characteristics for healthy live birth including: advanced reproductive age (average 37.2 years), an average of 5.8 years of infertility with 2.1 (range 1–9) previous IVF attempts, with only 5% having previously had a live birth from IVF. Despite these undesirable prognostic indicators, the overall RRM live birth rate was 32.1% (crude 18.4%). Women aged 35–38 had a live birth rate of 37.5% (crude 23.6%) and older women over 40 had a live birth rate of 27.4% (crude 16.0%). The average birth weight was 3374g (7lb 7oz) with 92% being born at 37+ weeks and no very low birth weight babies. There was only one twin pregnancy in the study population; the potential health care savings for avoidable multiple pregnancies in these patients was estimated at £205 672 (USD$284 915).

**Conclusions:** Patients who have already tried IVF can achieve comparable live birth outcomes with RRM compared to another cycle of IVF. RRM has a low risk of twin or multiple births, and very good neonatal outcomes with a potential cost savings to the health care system.

## Introduction

In the last four decades, Reproductive Medicine has evolved two divergent streams of care that have significant fundamental differences, most notably in their approach to infertility. Conventional reproductive medicine has developed treatments, such as controlled ovarian stimulation (COS), intrauterine insemination (IUI), *in vitro* fertilization (IVF), and intracytoplasmic injection (ICSI) that do not prioritize diagnosis or treating underlying conditions; rather the treatment largely bypasses underlying pathology (1). These approaches primarily focus on follicle stimulation protocols and refining ART (Assistive Reproductive Technology) such as IVF and ICSI, which are focused on achieving pregnancy independently of underlying pathology. In contrast, Restorative Reproductive Medicine (RRM) is medical, and surgical reproductive health care that aims to improve general and gynecological health and restore optimal reproductive function^1^,^2^. RRM operates on the principle that infertility is not a diagnosis but is the expression of underlying ill health conditions, often several, which if diagnosed and treated commonly result in restoration of normal reproductive function. When pregnancy is desired, conception can occur through regular intercourse.

A key component of RRM involves timing investigations and treatment with the help of a fertility chart, which is a chart of daily observations kept by the woman or couple, which additionally serves to monitor effects of treatment. There are a number of various cycle tracking methods that can provide the detailed information required for an RRM approach, these can be classified as Sympto-Thermal (Roetzer, Sensiplan, Serena), Sympto-Hormonal (Marquette, FEMM, Neo Fertility) and methods primarily based on observations of cervical fluid, such as the Creighton Model (CrM) and Billings Ovulation (BOM).

RRM treatment programs recognize that infertility is a complex disorder that more aptly fits a chronic disease model of care rather than episodic acute conditions, as is frequently practiced in reproductive medicine. In Ireland RRM treatment has been offered since 1998, through the clinics studied in this paper, and has been refined over time. The concept of infertility recognizing and managing infertility as a chronic disease was first proposed in 2011 (2). Characteristics of a chronic condition include: gradual onset, long latency period between onset and effect, the symptoms persists over a long duration of time (>3 months) with numerous factors contributing to the condition. Outcomes are often improved with multiple sustained interventions that involve patient education, empowerment and a component of self-management (3–5). Recognizing this, RRM treatment takes a comprehensive approach that identifies and corrects numerous physiologic and anatomic abnormalities contributing to the patient's infertility and encourage the patient to be an active participant in the patient-provider partnership (6, 7), rather than just a recipient of treatment as often happens in conventional care.

The patients experience this partnership in the initial phase of treatment, where they are taught how to chart their fertility cycle and are educated on reproductive physiology. The fertility chart plays a central role in an RRM program; not only for identifying an ovulatory event and timing intercourse, as is occasionally done in non-RRM treatment, but also as a critical tool for timing investigations and treatment. This chart is also a fundamental indicator of the impact of the various therapies on the reproductive cycle. The aim is to normalize chart parameters, including, but not limited to, length and pattern of bleeding, mucus quantity and quality, and luteal phase factors including lab and ultrasound indicators that are timed with the fertility chart. The woman and her partner are able to clearly see the impact of most treatments on her cycle using objective factors, such as the length of the luteal phase, mucus quantity, and quality and identification of her peak day (ovulatory event) as well as hormone levels: progesterone and estradiol, 7 days after ovulation (referred to as peak+7) (2). Thus the patients' experience forms the foundation for ongoing treatment.

Knowing and understanding the different models and principles which underlie RRM and prevailing reproductive medicine is important for patients who need to make a decision on how best to address their infertility. Previous published studies on RRM outcomes have demonstrated cumulative adjusted live birth rates using life-table analysis of 52.8% (crude rate 25.5) (8) to 66% (crude rate 38%) (9) in a mixed population with infertility. Conventional ART treatment such as IVF results in a relatively high number of preterm deliveries with a modest success rate. To evaluate the potential of RRM as an alternative for ART treatments, we analyzed live birth rates and pregnancy outcomes of RRM-treated patients with a history of one or more rounds of IVF.

## Methods

### RRM clinic

Between January 2004 and January 2010, the clinic operated in two sites in Ireland, Galway and Dublin. There were five physicians providing services during this time, all of whom were licensed for family medicine in Ireland. All had received training in RRM, specifically NaProTechnology using the Creighton Model fertility chart in the USA. Additionally they underwent extensive mentoring with the clinic medical director to be able to provide ultrasound services and further advanced RRM techniques.

### RRM assessment and treatment plan

The RRM treatment plan consists of three phases. Phase I is the investigative phase where patients learn specialized cycle tracking and where diagnostic testing is performed, including blood tests timed to the fertility (cycle) chart. During the duration of this study the Creighton Model charting system was used.

While establishing competence in fertility tracking, usually taking 2 months, patients undergo blood samples timed to their identified ovulatory event (peak day) and other laboratory investigations for both partners as indicated by their history and chart interpretation. Mid-luteal levels of progesterone and estradiol are measured in each treatment cycle with a goal of optimal levels 7 days after peak day (ovulation) of estradiol levels between 400–900 nmol/L and progesterone between 60 and 100pmol/L.

Upon completion of the investigative phase, usually in the third month, medications are prescribed to achieve cycle and hormonal optimization, Phase II. Details of this systematic multi-level process have previously been described (8). These may include medications for ovulation induction, cervical mucus normalization, as well as balancing follicular and luteal phase hormonal levels. Immunological, antibacterial, dietary, nutritional, surgical and psychological interventions may also be employed based on the individual patient's needs. Often confirmation of effective and complete follicular rupture is done by ultrasound in two separate cycles.

Phase III is the conception phase, which is characterized by cycles that demonstrate a normal fertility charting pattern with optimum levels of progesterone and estradiol on day 7 after ovulation and proven follicle rupture by ultrasound. The goal is to obtain 12 optimal cycles, which may take up to an additional 18 months to achieve.

Upon conception, patients' progesterone levels are monitored and progesterone support is provided throughout the pregnancy, most often with vaginal suppositories to maintain their levels in a normal range as determined by a published reference of progesterone levels in pregnancy (10).

### Patient inclusion criteria

All patients who presented between January 2004 and January 2010, with a history of infertility and previous IVF treatment were included if they proceeded beyond the initial consultation and began the treatment process. Outcomes were followed for up to 2 years after entry, up until January 2012. Patients who were pregnant within the first month after inquiring and receiving educational information were excluded, even if they required further hormonal monitoring and supplementation to assist with continuance of the pregnancy. We excluded two successful pregnancies that occurred beyond 24 months.

### Medical records and data collection

The data was routinely collected from patients during their initial and subsequent visits as recorded in the medical records. Additional information customarily collected through phone, mail or e-mail, and placed in the chart was also available for evaluation. Data required for this study was selected from the existing medical records and entered into a computerized database, with secondary verification of the information occurring as needed. Extracted data was anonymized with unique identifiers such that the data could only be verified manually by research staff.

### Data analysis

Evaluation of treatment for a chronic condition is best represented by a cumulative cohort approach, and not pregnancy rates per cycle, as is often done for IVF. The main outcome is live birth per couple. Subsequent pregnancies were not recorded. Secondary outcomes include conception and miscarriage rates, multiple births, birth weight and prematurity rates. Live birth rates were calculated using life table analysis.

### Ethics approval

The study protocol was reviewed and approved by the Galway Clinic Ethics committee. Data was abstracted from usual clinical data sources and patient anonymity was maintained, as such there was no requirement for written informed consent of participants.

## Results

### Characteristics of RRM patients and treatment outcome

This study includes 403 couples, who received RRM treatment after one or more IVF attempts between January 2004 and January 2010. The baseline characteristics of these patients are presented in Table 1. Only 22% had previously had a live birth; 5% (20 patients) through IVF. The mean age was 37.2 years. Patients had been trying to conceive an average of 5.8 years prior to starting RRM. Most had previously failed IVF attempts in the past, an average of 2.1 ART attempts per couple, with the range of 1–9 prior cycles as presented in Table 2.

**Table 1:** Characteristics of RRM patients with previous ART.

**Patients characteristics**	
Total (*n*)	403
Number of failed IVF attempts (mean [range])	2.1 (1–9)
Woman's age (mean years [range])	37.2 (23–45)
Prior years attempting to conceive (mean)	5.8
Had previous live birth (percent yes)	22 (5% IVF, 17% Natural)
Conceived always miscarried (percent yes)	25
Never conceived naturally (percent yes)	53

**Table 2:** Number of patients by previous ART attempts.

**Number of previous ART attempts**	**Number of patients**
1	154
2	136
3	71
4	23
5	13
6	3
7	2
9	1

Life Table analysis indicates an overall live birth rate of 32.1% with completed RRM treatment (Table 3). Adjusted proportions and complete RRM treatment were calculated based on up to 24 months of RRM treatment. Patient age, number of years trying or number of ART attempts were not significantly correlated with crude or adjusted proportional live birth rates (Table 4). Patients who conceived were monitored for hormonal levels and received progesterone support on average until 17.8 weeks of pregnancy, though 21% needed support beyond 24 weeks because of persistently low progesterone levels.

**Table 3:** Crude and Adjusted (Life Table) Conception and Live Birth Rates.

			**Conception**			**Live births**		
**Time interval (months)**	**Cumulative withdrawals from RRM (n “proportion”)**	**Starting at time interval (*n*)**	**Cumulative conceptions (*n*)**	**Crude proportion**	**Adjusted proportion**	**Cumulative live births (*n*)**	**Crude proportion**	**Adjusted proportion**
1-3	34 (8.4)	403	15	3.7	3.8	12	3.0	3.0
4-6	63 (15.6)	354	29	7.2	7.6	23	5.7	6.1
7-12	149 (37.0)	311	62	15.4	18.5	49	12.2	14.8
13-18	229 (56.8)	192	88	21.8	31.7	67	16.6	24.3
19-24	275 (68.2)	86	99	24.6	42.8	74	18.4	32.1

**Table 4:** Live Birth Rates by age, time spent trying to conceive and previous ART attempts.

**Couple category**	**Couples (*n*)**	**Live births (*n*)**	**Crude proportion**	**Adjusted proportion**
All couples	403	74	18.4	32.1
**WOMAN'S AGE (YEARS)**				
< 35	87	15	17.2	28.0
35–37	127	30	23.6	37.0
38–40	108	16	14.8	28.1
>40	81	13	16.0	27.0
**TIME SPENT ATTEMPTING TO CONCEIVE (YEARS)**				
1–3	77	19	24.7	40.7
>3–6	188	31	16.5	23.7
>6–9	92	20	21.7	45.7
>9	46	4	8.7	20.1
**PREVIOUS ART ATTEMPTS (*****n*****)**				
1	154	31	20.1	33.4
2	136	22	16.2	28.1
3+	113	21	18.6	33.0

### Fewer multiples and higher birth weight with RRM

**Figure 1:**
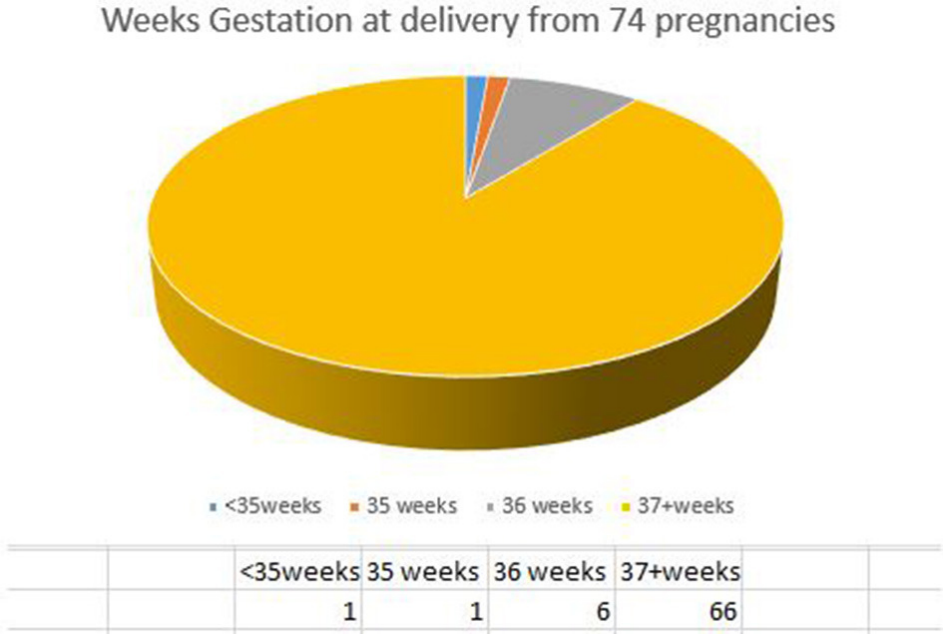
Gestational age at birth for RRM pregnancies after IVF.

Besides probability of live birth, other birth outcome parameters like the percentage of multiples and weeks of delivery are very important outcome criteria for fertility treatments. Birth outcome parameters of all patients on RRM treatment are presented in Table 5. The average birth weight was 3374g (7lb 7oz); from all live births only 1.4% were twins, with no triplets conceived. None of the live births demonstrated a very low birth weight (< 1500g). The overall percentage of live births with a low birth weight (< 2500 g) was 6.8% (5 out of 74), but only 5.5% (4 out of 73) for singletons. For the one twin pregnancy, both babies had a low birth weight of 2070 (4lb 9oz) and 2240g (4lb 15oz) respectively. The percentage of preterm deliveries from the singleton babies was 8.2% (6 out of 73), while the twin pregnancy was delivered by elective C-section at 36 6/7 weeks, as was the practice at the time (Figure 1).

**Table 5:** Birth outcome of RRM.

	**RRM (all patients)**
Years treated	2004–2010
Live birth deliveries	74
**MULTIPLES**	
Twins-mean-%	1.4
Triplets or higher	0
**LOW-BIRTH WEIGHT (**<**2500 GR)**^#^	
Singletons-%	5.5
**VERY LOW BIRTH WEIGHT (**<**1500 GR)**^#^	
Singletons-%	0
**PRETERM (**<**37 WEEKS)**^#^	
Singletons-%	8.2^*^

# *One twins elective C-section at 36 weeks and 6 days with birth weights of 2.1 and 2.2 kg*.

* *From the six preterm singleton deliveries there were four at 36 weeks, one at 35 weeks and one at 33 weeks. Unknown birth weight and weeks of delivery for 2 pregnancies*.

## Discussion

The decision to pursue or continue fertility treatment is not an easy one for patients. Many factors play a role in this decision making process, most notably the likelihood of successful treatment and the risk to mother and baby. For those patients who choose to pursue treatment many are encouraged to undergo IVF, with or without ICSI as the “presumptive gold standard” of infertility treatment, particularly patients with long-standing infertility or those who are older than 35. Many infertility experts counsel patients to consider more than one IVF cycle to achieve a reasonable overall live birth rate (11). Many factors, including initial response to the treatment cycle, patient age and finances, must be considered in this process (12). The stress of undergoing IVF is substantial for couples (13, 14) and many welcome alternatives that are less invasive with comparable outcomes in live birth rates and fewer complications.

Previous publications on RRM outcomes have demonstrated an overall success rate of 52.8–66% using lifetable analysis (8, 9). In this paper, the overall success rate in a population of patients with a very poor prognosis due to advanced maternal age (37.2), long-standing infertility (5.8 years) and, on average, 2.1 previous IVF treatments is 32.1% by life table analysis (crude rate 18.4%). To compare these results with repeated IVF attempts is complicated.

Data linking each IVF cycle to the patient on repeat IVF cycles is not readily available in most ART databases. The Human Fertilisation and Embryology Authority (HFEA) collects UK data on IVF outcomes; in the HFEA 2011 trends and figures publication they report that 2/3 of women who undergo IVF are under 37 and that “…women who have the best chance of becoming pregnant are usually those aged 37 and under, who are on their first or second attempt at IVF” (p.19) (30). Most RRM patients in this clinical setting, however, are older with many years of infertility; 47% in the study were older than 38, and 20% were 41 and older.

Supplemental data reported in a study by Luke et al. (15) provides some very basic comparisons for this group of older patients. This is a large study of over 200,000 patients where live birth rates were calculated for different age groups for an increasing number of linked IVF attempts. The conditional rates are calculated on the premise of no previous successful IVF attempts in the group, while the cumulative rates calculated include the success rates for each previous IVF cycle. The comparison is not straightforward; the demographics, though, are similar, thus, RRM patient outcomes with previous IVF can be compared to the conditional rates in the supplemental data by age category. For those 38–40 years old with 2 failed IVF attempts, a live birth rate of 17.5% was found after an additional IVF cycle, while these percentages dropped further to 9.5 and 3.6% for patients of respectively 41–42 and 43+ years old. With RRM, 38–40 year old women demonstrated adjusted life birth rates of 28.1% (crude 14.8%), while this percentage remained high in patients over 40 with an adjusted rate of 27.0% (crude 16.0). After three failed IVF attempts, patients may attempt even more IVF cycles, however with live birth rates below 10% per IVF attempt for patients above 40 years old, RRM may provide a more suitable option for these patients.

This paper highlights some of the challenges that exist when trying to compare RRM and IVF. RRM approaches infertility as a symptom of a chronic condition, with a multitude of factors contributing to the issue. Treatment is aimed at addressing each of these, with continued improvement each cycle as the goal. The patient experiences an objective measurable improvement in chart and lab parameters, most notably progesterone and estradiol 7 days post-ovulation, as well as ultrasound investigations. IVF, however, is designed as a single acute intervention that is inherently a highly invasive method of circumventing underlying pathophysiology. Patients most often need to take medication to suppress natural ovulation and create supraphysiologic ovulatory conditions. This is followed by surgical egg retrieval and subsequent lab facilitated fertilization or freezing, with ensuing embryo transfer, an additional invasive procedure. The measurement of IVF success is usually pregnancy or live birth rates per cycle of treatment. In this paper, comparison is made with a study that analyzed IVF outcomes over time and with repeat cycles, providing some crude approximation of a comparative longitudinal approach; however, this comparison is necessarily simplified. Further work on models to better compare these methods is needed.

Several large studies on IVF have raised concerns about the risks and sequelae of multiple pregnancies and perinatal complications including low birth weight and prematurity (16, 17). Despite significant efforts to reduce the rates of multiple births, including the use of single embryo transfer and fetal reduction (18), they remain a substantial concern today. Treatment options for infertility that have lower rates of prematurity and less babies born with a low birth weight are appealing for couples and the broader health care system. With RRM infants born with low birth weight and prematurity from higher order pregnancies (19) are avoided. In this study we observed only one twin pregnancy (twin rate of 1.4%) and low rates are reported in other studies as well (8, 9). Besides the issues associated with multiple births, recent research indicates that singleton pregnancies born through IVF are also at increased risk for prematurity and low birth weight (20, 21). There is research to suggest that this risk is not solely a result of IVF, but may be associated with infertility (22). Furthermore, older women, by reason of their age alone, would also be considered at higher risk (23, 24) for having babies born prematurely and with low birth weights. It is interesting then that the rates of prematurity and low birth weight among RRM live births, of which 20% of women are over 40 years old, are similar to those of the general population of Irish women of all reproductive ages (Ireland premature birth rate 6%, RRM rate 5.5%; Ireland low birth weight rate is 6%, RRM 8.2%)^3^. This would suggest that restoring reproductive potential in a comprehensive way, as practiced with RRM, may largely overcome this problem.

The success of RRM treatment is likely due to several factors. Unlike IVF, RRM focuses on the correction of identified abnormalities in reproductive physiology prior to conception, in particular ensuring appropriate ovarian hormonal production, effective ovulation and high quality cervical mucus production to facilitate sperm transport. Conception occurs from natural intercourse. This restorative approach likely addresses much of the underlying pathophysiology that contributes to pregnancy complications and premature delivery.

RRM also ensures that after conception progesterone is closely monitored and supplemented with human identical progesterone, according to a standardized protocol (10). Studies have demonstrated a significant reduction in the rate of premature delivery with standard dose progesterone supplementation in high-risk patients, continued into the third trimester (25–27). In RRM, the monitoring of serum progesterone levels is used as part of an evaluation for identifying patients at risk of pregnancy loss or premature delivery and treatment is provided as necessary to maintain levels according to a standard graph. This may account for some of the reduction in premature delivery; however, the fact that on average progesterone support was provided for only 17.8 weeks suggests that correcting the pre-conceptive and early pregnancy hormone levels, particularly progesterone, contributes to this outcome. 21 pregnancies (28%) continued progesterone support beyond 24 weeks due to suboptimal levels, indicating a likely benefit of continuing progesterone treatment into the 3rd trimester in progesterone deficient patients. It is believed that progesterone may have a significant immunologic role to play in pregnancy and some preliminary research shows that it may suppress the embryotoxic action of the complement system, facilitating healthy early embryo development (28). Further research exploring the correlation between pre-conceptive, conceptive and pregnancy hormone levels and pregnancy outcome is necessary.

The implications of these results are potentially far-reaching. RRM may provide an effective, safe alternative for many couples with infertility, even those who have already undergone IVF, with the possibility of achieving good live-birth outcomes and substantially reducing prematurity and low birth weights. These may not only reduce health complications for the affected children, but could potentially result in significant cost savings as well.

A 2006 British study calculated the costs at £9,122 for a twin pregnancy and £32,354 for a triplet pregnancy in 2000-2001 (29). If these cost estimates were applied to the patients in this study who had a live birth, but had undergone IVF with a twin rate of 25% (19 twins) and a triplet rate of 1% (1set of triplets), as noted by HFEA in their 2006 IVF statistics, the cost to the health care system for avoidable multiple pregnancies would have been calculated at £205,672 ($US284 915). This represents a significant expense, not only to the patients and their families, but also to the health care system. Higher order pregnancies from ovarian stimulation and intrauterine insemination are not included in these figures, but may represent a significant contribution to multiple births and would be also avoidable with RRM and restorative ovarian stimulation.

Much more research is needed in the field of RRM, particularly direct comparison to patients undergoing IVF/ICSI. However, patients who are deciding on their next steps after failed IVF treatment can be reassured that RRM outcomes provide them at least equal chance of attaining biologic parenthood, without the ensuing risk to their babies and with cost savings to their family and the healthcare system.

## Author contributions

TG, KA, PB, and TP: Acquisition, analysis and interpretation of the data. TG and KA Statistical Analysis. TP: Drafted the manuscript. TG, KA, PB, and TP: Reviewed and modified the manuscript.

### Conflict of interest statement

The authors declare that the research was conducted in the absence of any commercial or financial relationships that could be construed as a potential conflict of interest.

## Footnotes

^1^https://www.frontiersin.org/research-topics/6166/current-research-in-restorative-reproductive-medicine

^2^https://restorative-reproductive-medicine.com

^3^https://www.hse.ie/eng/services/news/media/pressrel/newsarchive/archive15/mar15/perinatalreport.html
